# The mPED randomized controlled clinical trial: applying mobile persuasive technologies to increase physical activity in sedentary women protocol

**DOI:** 10.1186/1471-2458-11-933

**Published:** 2011-12-14

**Authors:** Yoshimi Fukuoka, Judith Komatsu, Larry Suarez, Eric Vittinghoff, William Haskell, Tina Noorishad, Kristin Pham

**Affiliations:** 1 Institute for Health & Aging, Department of Social and Behavioral Sciences, University of California, San Francisco, 3333 California Street, Suite 340, San Francisco, CA 94143, USA; 2 Institute for Health & Aging, University of San Francisco, San Francisco, CA, USA; 3 Information Services Unit, University of California, San Francisco School of Medicine, San Francisco, CA, USA; 4 Department of Epidemiology & Biostatistics, University of California, San Francisco, CA, USA; 5 Stanford Prevention Research Center, School of Medicine, Stanford University, Palo Alto, CA, USA

## Abstract

**Background:**

Despite the significant health benefits of regular physical activity, approximately half of American adults, particularly women and minorities, do not meet the current physical activity recommendations. Mobile phone technologies are readily available, easily accessible and may provide a potentially powerful tool for delivering physical activity interventions. However, we need to understand how to effectively apply these mobile technologies to increase and maintain physical activity in physically inactive women. The purpose of this paper is to describe the study design and protocol of the mPED (**m**obile phone based **p**hysical activity **ed**ucation) randomized controlled clinical trial that examines the efficacy of a 3-month mobile phone and pedometer based physical activity intervention and compares two different 6-month maintenance interventions.

**Methods:**

A randomized controlled trial (RCT) with three arms; 1) PLUS (3-month mobile phone and pedometer based physical activity intervention and 6-month mobile phone diary maintenance intervention), 2) REGULAR (3-month mobile phone and pedometer based physical activity intervention and 6-month pedometer maintenance intervention), and 3) CONTROL (pedometer only, but no intervention will be conducted). A total of 192 physically inactive women who meet all inclusion criteria and successfully complete a 3-week run-in will be randomized into one of the three groups. The mobile phone serves as a means of delivering the physical activity intervention, setting individualized weekly physical activity goals, and providing self-monitoring (activity diary), immediate feedback and social support. The mobile phone also functions as a tool for communication and real-time data capture. The primary outcome is objectively measured physical activity.

**Discussion:**

If efficacy of the intervention with a mobile phone is demonstrated, the results of this RCT will be able to provide new insights for current behavioral sciences and mHealth.

**Trial Registration:**

ClinicalTrials.gov#:NCTO1280812

## Background

Physical inactivity is associated with an increase in cardiovascular disease, Type II diabetes, hypertension, certain types of cancer, and obesity [1-3]. The National Physical Activity Guidelines recommend at least 150 minutes a week of moderate-intensity physical activity [4]. However, approximately half of American adults, particularly women and minorities, do not meet this recommended physical activity recommendation [5].

Mobile communication technologies are widely available; the majority of adults in the United States own a mobile phone [6]. Mobile phones have the advantage of being portable and easily accessible. These are qualities that make them powerful tools for delivering physical activity interventions. Despite this considerable potential, the majority of mobile phone based physical activity intervention studies have had a relatively small sample size, have evaluated only the short-term effects of the intervention, and have been conducted without a proper control group [7-9]. Thus, it is important to systematically investigate whether mobile phone interventions work effectively, and over what period of time.

In order to better understand the feasibility and acceptability of a mobile phone based physical activity intervention, we conducted a three week pilot study on physically inactive women in the San Francisco Bay Area. The results of both quantitative and qualitative analyses of this pilot study have been published elsewhere [8,10,11]. In the pilot study, sedentary women wore a pedometer and were issued a mobile phone loaded with a custom-designed physical activity mobile phone application. In summary, this pilot study had a relatively high adherence rate for use of all components of the intervention; the pedometer, physical activity diary and response to daily messages. In addition, the women in the pilot study significantly increased their objectively measured steps over the three week observation period [8].

After completion of the pilot study, some improvements were made to the mobile application in order to prepare for a full-scale randomized control clinical trial intended to test the efficacy of the mobile phone based physical activity intervention among sedentary women. The final usability test was further conducted with six women who were unfamiliar with the proposed study prior to launching a full-scale randomized controlled clinical trial (RCT) of our **m**obile phone based **p**hysical activity **ed**ucation (mPED) program.

### Study aims

The aims of this RCT are threefold: 1) to assess the efficacy of the 3-month mobile phone and pedometer based physical activity intervention on increasing physical activity compared to the control group; 2) to compare the efficacy of the 6-month maintenance intervention-PLUS to the 6-month maintenance intervention-REGULAR; and 3) to explore the role of potential mediating factors (self-efficacy for physical activity, social support, and barriers for physical activity) and moderating factors (body mass index and age) on changes in outcomes at 3, 5, 7 and 9 months. The primary outcome measure for the first two aims is the total steps per day as measured by the Omron Active Style Pro HJA-350IT (triaxial accelerometer) pedometer.

## Methods

### Study design

**Figure 1:**
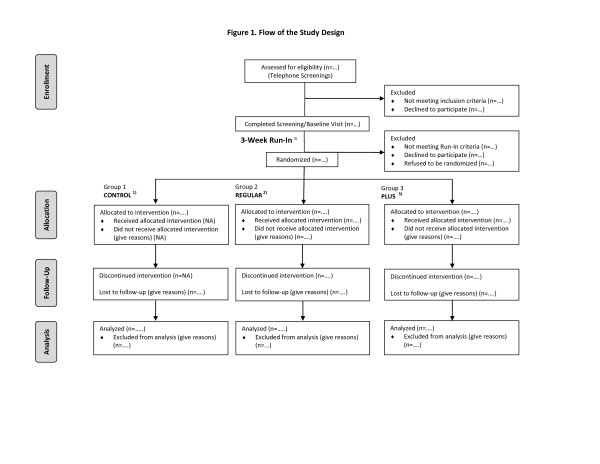
**Flow of the study design**. ^0) ^3-week run-in: To be randomized, participants must successfully complete a 3-week run-in period (defined as at least 80% adherence rate in using daily message and diary and at least eight hours a day for ≥ 80% of the run-in period and completed a fasting blood test in a research lab). ^1) ^CONTROL: Pedometer only for 9-months, no physical activity intervention, ^2) ^REGULAR: 3-month mobile phone and pedometer based physical activity intervention and 6-month pedometer maintenance intervention, and ^3) ^PLUS: 3-month mobile phone and pedometer based physical activity intervention and 6-month mobile phone diary maintenance intervention.

This is a randomized controlled clinical trial with three arms; 1) PLUS (3-month mobile phone and pedometer based physical activity intervention and 6-month mobile phone diary maintenance intervention), 2) REGULAR (3-month mobile phone and pedometer based physical activity intervention and 6-month pedometer maintenance intervention), and 3) CONTROL (pedometer only, no intervention will be provided). All groups receive a pedometer to use for nine months. (See Figure 1)

### Ethical approval

The study protocol including informed consent form, study questionnaires, educational and recruitment materials has approved by the University of California, San Francisco Committee on Human Research (Institutional Review Board) and the Data and Safety Monitoring Board (DSMB). All potential participants will receive a copy of the informed consent electronically or by mail after successful completion of the telephone screening. This consent will be reviewed and signed in the research office before enrollment in the study.

### Participants

A total of 192 physically inactive women who meet all inclusion criteria and successfully complete a 3-week run-in period will be randomized into one of the three groups. Physically inactive women will be recruited from the San Francisco Bay Area through the use of local newspaper advertisements, Craigslist (a classified advertisement website), Facebook, and flyers posted at local hospitals, medical clinics, dental clinics, community centers, and supermarkets, with the intent of recruiting a diverse and representative sample.

### Inclusion/Exclusion criteria

Inclusion criteria are: 1) sedentary lifestyle at work and/or during leisure time, as assessed using the Stanford Brief Physical Activity Recall Survey [12], Physical Activity History Questionnaire [13] and baseline average daily steps measured during the run-in period; 2) intent to be physically active; 3) female, age 25 - 69; 4) access to a home telephone or a mobile phone; 5) speak and read English; and 6) body mass index (BMI) between 18.5 - 43.0 kg/m^2^. The exclusion criteria are: 1) known medical conditions or other physical problems that need special attention in an exercise program (e.g., history of myocardial infarction, angioplasty, or angina, admission to the hospital for evaluation of chest pain, use of nitroglycerin to treat angina, uncontrolled hypertension, diabetes mellitus with insulin treatment, osteoporosis, or under active treatment for cancer); 2) planning an international trip during the next four months (subjects are required to upload mobile phone data to a server daily, and will not be able to do so from abroad); 3) pregnant/delivered a baby during the past six months; 4) known severe hearing or speech problem; 5) history of eating disorder (e.g. binge eating disorder, bulimia nervosa, anorexia nervosa); 6) in recovery from substance abuse; 7) currently participating in lifestyle modification programs or research studies that may potentially confound the results of the study; and 8) history of bariatric surgery or plans for bariatric surgery in the next twelve months. We will not exclude women who have never used a mobile phone or are not current mobile phone users.

### Screening/Baseline visit and run-in period

All potential subjects will be screened by telephone and eligible subjects will be invited to attend a screening/baseline visit. After obtaining a written informed consent, baseline data will be gathered to determine eligibility. Eligible participants will be issued a mobile phone and pedometer; training will be provided to insure participants can successfully use both devices. During the 3-week run-in, the pedometer will be set to record and store physical activity (e.g. steps), but no display of steps will be visible (only date and time will be visible) on the pedometer. A run-in mobile phone application has been created specifically for this phase of the study; it is designed to mimic the intervention application without any content to encourage or support increasing physical activity.

The run-in period has two purposes: 1) to determine the baseline average daily steps and 2) to determine if the participant will be able to comply with the requirements of the study. In order to be eligible for randomization, participants must successfully complete a fasting blood test at a research lab in addition to completing the run-in period with at least an 80% adherence rate in each of the following: 1) daily message responses, 2) daily activity diary responses, and 3) pedometer wearing time of at least 8 hours per day.

### Randomization

One hundred and ninety-two women will be randomly assigned in a 1:1:1 ratio to the PLUS, REGULAR or CONTROL groups. Permuted-blocked randomization will be used to ensure that the number of participants in the three treatment groups is close to our goal of an exact 1:1:1 ratio. Block size will vary randomly from three to nine in a schedule that is not known to investigators and research staff. The data management staff will prepare a set of sealed, opaque envelopes, numbered consecutively, and containing the study group assignment.

### Blinding

Due to the nature of the mobile phone intervention, blinding the participant, research staff, and investigator is not possible. In order to minimize expectation bias, participants are informed only that we are testing two different physical activity interventions (pedometer intervention versus pedometer plus mobile phone application intervention). Participants who are randomized into the PLUS and REGULAR groups are blinded to their maintenance intervention (PLUS or REGULAR) assignment until they complete the 3-month mobile phone and pedometer based intervention.

### Physical activity interventions

The physical activity interventions were designed based on the Social Cognitive Theory [14]. There are two components to the physical activity intervention: 1) a brief face-to-face physical activity intervention, and 2) the 3-month mobile phone and pedometer based physical activity intervention. All women randomized into the PLUS and REGULAR groups will receive the 3-month mobile phone and pedometer physical activity intervention in an identical fashion.

#### Brief face-to-face brief intervention

Upon randomization into a 3-month mobile phone and pedometer physical activity intervention group, each woman in the PLUS and REGULAR groups will be given a face-to-face interactive session by trained research staff. The face-to-face intervention is a structured interview containing seven domains: 1) overview of the physical activity program and tailored short and long-term goal setting; 2) education about duration and intensity of brisk walking and the health benefits of exercise; 3) identification of barriers to increasing physical activity and development of strategies to overcome these barriers; 4) value and identification of social support while increasing physical activity; 5) relapse prevention; 6) education about healthy diet and weight maintenance; and 7) physical activity safety. The face-to-face intervention is designed to be interactive and actively elicits the participant's participation. An individualized written physical activity plan will be developed during the face-to-face intervention. The trained research staff will revisit and revise the plan at the 1.5- and 3-month visits.

#### Three-month mobile phone and pedometer intervention

Two different mobile phone applications were created for this RCT: the trial application (the physical activity intervention) and a content neutral run-in application. The physical activity application used during the 3-month mobile phone and pedometer intervention phase will have three components 1) the daily message/video clip, 2) daily mobile phone diary, and 3) other functions ("Talk to us", "Summary", and "Help" menus). The run-in application will mimic the format of the trial application, but will not contain any content to support a physical activity program. The run-in and trial mobile phone applications were developed to run on two different mobile phone platforms: the Java 2 platform, Micro Edition (J2ME) and the iOS (iPhone) platform. If the participant has a compatible mobile phone or an iPhone, the application is installed on their personal phone. If participants do not want to install the application on their phone or do not have a compatible mobile phone, we provide a Motorola RAZR v3xx or a Pantech LASER study mobile phone, along with voice, text messaging (short message service) and data plans.

#### Daily messages/video clips

The daily messages/video clips of the trial application reinforce the seven domains addressed in the brief face-to-face intervention; the domains are divided into twelve weekly themes (see Table 1). In order to assure the comprehensibility of the daily messages, Flesch-Kincaid Reading Grade Level (RGL) was used to assess the readability of the daily messages [15]. The daily prompts had a RGL of 4.0 or a 4th grade reading level. A pre-programmed daily message or video clip will be automatically sent at a predetermined time between 11 a.m. to 3 p.m. The daily message will be accessible on the mobile phone until 7 p.m., if no reply is made by this time, the daily message will disappear from the phone. An automated text message is generated to alert participants that the daily prompt has arrived. Each daily prompt begins with a message from the research staff, followed by a question relevant to the message. For example, on Day 5 of Week 4, subjects receive the following daily message: *'Have you let everyone around you know that you are trying to become more active so they can help you meet your goal?' *"No" or "Yes" is selected by pushing the keypad. If "No" is selected, the next screen will display "*Let others know your physical activity goal*." If "Yes" is selected, the next screen will display *"Nice work!" *It takes only 1 one to two minutes to complete the daily prompt each day.

**Table 1:** Weekly themes for mobile phone application

Week 1	Monitoring daily steps/physical activity goal setting
Week 2	Identifying barriers and benefits of physical activity

Week 3	Adding a 10 minute walk

Week 4	Increasing social support for physical activity

Week 5	Building physical activity into daily activities

Week 6	Identifying community resources

Week 7	Recognizing and addressing lapses in physical activity

Week 8	Healthy diet and lifestyle tips

Week 9	Managing stress

Week 10	Maintaining motivation

Week 11	Exploring and adding new physical activities

Week 12	Wrapping-up and looking forward

#### Physical Activity diary

In our three week pilot study, the average timing of mobile diary use was approximately 9:30 pm [8], but some subjects used the mobile diary even before 8 p.m.

In this RCT, the physical activity diary will be accessible after 7 p.m. If no entry is made by 8:30 p.m., an automated text message is generated as a reminder for the participant to record the total number of steps per day and the types, and duration of physical activities.

The mobile phone diary program was designed so subjects have to answer questions sequentially. For example, when "Diary" is selected, the first question is: *"Did you wear a pedometer all day today, except for showering, swimming, or sleeping?" *If the answer is "Yes," the woman will be directed to enter the number of steps taken that day. To increase accuracy of data entry, a range of steps (1000 and 35,000) was programmed in advance. The participant will immediately receive a daily step histogram showing the daily step count, enabling her to monitor/visualize her progress. If the answer is "No" (i.e. - she didn't wear the pedometer), she will be asked to select the reason why and will receive suggestions based on her answer.

The next question is, "*Did you do more than 10 min of physical activity since midnight last night?" *If "Yes", she will be prompted to select the type and to enter the total number of minutes of physical activity. This entry generates a message providing immediate feedback based on the total number of minutes entered. For example, thirty minutes of physical activity generates the response, "Excellent job!"

#### Other functions

In addition to daily message (intervention) and mobile phone diary, the trial application includes "Summary", "Help", "Talk to us", and "Weekly goals" menu options. The "Summary" menu includes the material provided in the face-to-face intervention, the "Help" tab lists the research office contact information, and the "Talk to us" function allows the subject to directly send a text message to researchers from the application.

The activity goal is automatically updated each week and displayed in the "Weekly goals" tab on the application home screen. The mobile phone trial application automatically determines weekly daily step goals (short-term goal) by calculating a 20% step increase per week from the subject's average baseline daily steps. The long-term goal for all participants is to take at least 10,000 steps per day, seven days a week and to maintain this level of physical activity for the duration of the study.

### Physical activity maintenance

There are two different 6-month maintenance interventions, REGULAR (pedometer only) and PLUS (pedometer plus mobile phone physical activity diary). A 6-month maintenance period follows the 3-month mobile phone and pedometer based physical activity intervention; participants will be randomly assigned to one of two different maintenance interventions at the randomization visit. The maintenance intervention-REGULAR group will use the pedometer daily and the maintenance intervention-PLUS will use the pedometer and physical activity mobile phone diary daily. The purpose of the two different maintenance intervention groups is to gain insight into the "dose-response" to self-monitoring and feedback. In other words, is keeping a mobile phone diary and pedometer for 6-months a better physical activity maintenance method than a pedometer alone? The subjects in the control group continue using a pedometer for these six months of the study. The subjects in all three groups will be asked to bring back the pedometer to a research office to download the pedometer data at 5, 7 and 9 months. At the 9-month visit, participants will return all pedometers and study mobile phones (if there is any) to the research office. All participants will receive a follow-up telephone call at 12 months to assess their level of physical activity using the 7-Day Physical Activity Recall Questionnaire.

### Control group (pedometer-only group)

Participants randomized to the control group will continue using the pedometer without the intervention. Following the randomization visit, the pedometer settings will be changed so that the total steps are visible. Although providing a pedometer for the control group may temporally increase physical activity from baseline, we expect physical activity will not continue to increase in this group after the first few weeks of the study.

### Study measures

The primary outcome measure is the total steps per day as measured by the Omron Active Style Pro HJA-350IT (triaxial accelerometer) pedometer. This pedometer displays daily steps, automatically resets the step count at midnight and allows participants to view the past seven days of step counts. Data from the most recent 150 days performance are automatically stored and can be directly downloaded to a computer. The criterion for acceptable pedometer data is that the downloaded data must show that the participant wore the pedometer at least eight hours per day, and at least four days per week.

Secondary outcome measures are Intensity of Physical Activity per Day, and the Seven-day Physical Activity Recall (PAR) [16]. The Omron Active Style Pro HJA-350IT can measure activity intensity over a 60 second epoch and estimate the metabolic equivalent (METs) of the task. In this study, aerobic steps are defined as ≥ 10 min of continuous steps ≤ 3 METs (approximately > 120 steps/min). Total minutes spent in moderate (3-6 METs) and vigorous activity (≥ 6 METs) during the previous week will be used as a secondary physical activity outcome measure. An interviewer-administered PAR will be used to assess physical activities performed during the week preceding the randomization, 3 and 9-month follow-up visits and 12-month follow-up phone call.

Other study measures will be the Self-Efficacy for Physical Activity Survey [17], Social Support and Exercise Survey [18], 12-Item Short-Form Health Survey (SF-12) [19], Barriers to Being Active Quiz [20], and the Center for Epidemiological Studies Depression Scale [21].

Sociodemographic characteristics (ethnicity, marital status, date of birth, education level, employment status, annual household income, marital status, number living in the home, employment status, and health insurance status), medical history (hypertension, hyperlipidemia, etc.), current smoking status, current alcohol intake, and current medications (name and dosage) will be collected at the screening/baseline visit. All subjects will undergo a physical exam consisting of the following measures: resting blood pressure, body weight in kilograms, height in centimeters, waist and hip circumference in centimeters. For the height, weight, hip and waist circumference measurements, patients will change into a hospital gown and remove their shoes. Body mass index will be determined by dividing the body weight (kg) by the square of the height (m^2^).

A fasting blood specimen collected at baseline and at seven months will include the following tests: hemoglobin A1c, blood glucose, total cholesterol, high-density lipoprotein (HDL) cholesterol, low-density lipoprotein (LDL) cholesterol, and triglycerides.

Adverse events (AEs) will be monitored and recorded throughout the study period. An adverse events checklist will be completed at the 1.5, 3, 5, 7 and 9-month visits and the 12-month follow up call. In addition, if a subject in either intervention group has not used their mobile phone for more than seven days consecutively, they will be contacted by the research staff to assess potential adverse events (AEs). All reported AEs will be evaluated by the Principle Investigator and reported to the Data and Safety Monitoring Committee and the institution's Committee on Human Research.

### Data management and security

There will be three modes of data collection in this research study: in-person, pedometer and mobile phone. All questionnaire data instruments will be collected on machine-readable data forms based on the Cardiff Teleform software system. Completed teleforms will be faxed to the San Francisco Coordinating Center where a server using optical character recognition (OCR) technology will verify and store the data in the study database on a secure Microsoft SQL server. A standardized procedure will convert the SQL data to SAS data.

Data from participant's pedometers will be downloaded at the research office and electronically transferred to the San Francisco Coordinating Center. Data from the mobile phone is collected in real-time on the mobile phone's memory card, time-stamped and stored for transmission over a wireless network to a secure server. A secure online data management service, SugarCRM^®^, will be used to capture the mobile phone data. Each night, after using the physical activity diary, the participant will be prompted to exit the application, at this time the data will be transmitted to SugarCRM^®^. These data will all be encrypted. If the wireless network is unavailable, data will be stored on the mobile phone and transmitted when the wireless network next becomes available. A daily report is generated by SugarCRM^® ^and reviewed daily by the research team to monitor compliance with the mobile phone trial application. Both the mobile phone and data storage will Health Insurance Portability and Accountability Act (HIPPA) compliant. All participants will be assigned a study identification number immediately after enrollment; this identifier will be used on all study data.

### Statistical analysis and power calculation

The primary analyses will be by intention-to-treat, without regard to adherence to intervention protocols. We will use linear mixed models to compare trends in the primary and secondary endpoints. To avoid inflating the type-I error rate, differences in the maintenance period will be tested only if the intervention is shown to be successful during the trial. If dropout is substantial, sensitivity analyses will be conducted in which we multiply impute missing outcome data under the conservative assumption that exercise levels fall back to their baseline levels after study dropout. The validity of the randomization will be assessed by comparing randomized groups on characteristics measured before each randomization. If potentially confounding imbalances are found, we will adjust the between-group analyses for potential confounders of treatment assignment.

#### Sample size and power

We assumed that the net effect of the intervention would be to increase average daily steps by 2200, based on our pilot data and recently published review papers [22,23]. During the maintenance period, we assumed that the difference between PLUS and REGULAR maintenance groups would be 1100 steps. We used data from the Woman's Exercise Injuries: Incidence and Risk Factors (WIN study) [24] to estimate the standard deviation and within subject correlation of the primary outcome. Finally, we assumed that 88% of participants would remain in follow-up at 3 months and that 75% would remain in follow-up at 9 months. To provide 90% power to detect a between-group difference of 1100 steps per day in the maintenance period, we will require that 114 women be randomized in a 1-1 ratio to the PLUS and regular maintenance groups. The overall sample size of 192 for the trial will provide more than 95% power to detect a between-group difference of 2200 steps, and more than 90% power for net differences as small as 1000 steps.

## Discussion

The purposes of this randomized controlled trial are to test the efficacy of a 3-month mobile phone and pedometer based physical activity intervention and to compare two different 6-month maintenance interventions. In this RCT, the mobile phone serves multiple purposes: the means of delivering the physical activity intervention: sets individualized weekly physical activity goals: provides self-monitoring (diary); provides immediate feedback, social support, and real-time data capture. To our knowledge, this is the first mobile phone based physical activity intervention to incorporate all functions listed above. It is also the first to use a RTC design to test the efficacy of such an intervention in a diverse sample of physically inactive women. This RCT design has several unique features and may add new knowledge to current behavioral sciences. One of the strengths of this RCT is the length of mobile phone use. Specifically, a 3-month mobile phone and pedometer based intervention, followed by a 6-month active maintenance intervention (PLUS or REGULAR) exceeds the length of most previous trials [7-10,25-32]. In addition, by utilizing two different physical activity maintenance interventions, this study has the potential to make a major contribution to our understanding of maintenance strategies in terms of a dose-response with or without a mobile phone diary component. Lastly, the combination of objectively measured downloadable physical activity (e.g. steps, duration, and intensity) data and the time-stamped mobile phone data will allow the research team to better understand the efficacy and mechanism of the physical activity mobile phone intervention among physically inactive women.

A few limitations of this study need to be acknowledged. First, although J2ME and iPhone applications are available in this RCT, the physical activity intervention is incompatible for all mobile phone platforms. If a participant has an incompatible mobile phone, she will be required to use a study mobile phone for the duration of the study intervention in addition to her own mobile phone. This may potentially reduce adherence to the mobile phone based intervention over a longer period of time, although our pilot study showed excellent adherence over a three week period. A second limitation of this study is that Omron Active Style Pro HJA-350IT (triaxial accelerometer) pedometer is unable to capture and record physical activity data from bicycling, swimming or yoga. Therefore, we will also use the Seven-Day Physical Activity Recall questionnaire to capture these activities at 3, 7, 9 and 12 months. In addition, the mobile phone diary will include a question as to how long subjects engage in these activities, if applicable.

## Abbreviations

AE: Adverse event; BMI: Body mass index; CES-D: Center for epidemiological studies depression scale; DSMB: Data safety and monitoring board; HDL: High density lipoprotein; HIPPA: Health insurance portability and accountability act; iOS: i operating system; IRB: Institutional review board; J2ME: Java 2, micro edition; Kg: kilogram; LDL: Low density lipoprotein; MET: Metabolic equivalent of task; mHealth: Mobile health; mPED: Mobile phone based physical activity education; OCR: Optical character recognition; PAR: Seven-day physical activity recall; RCT: randomized controlled trial; RGL: Reading grade level; SAS: Statistical analysis system; SF-12: 12-item Short-form health survey; SPSS: Statistical package for social science; UCSF: University of California, San Francisco; WIN: Woman's Exercise Injuries: Incidence and Risk Factors.

## Competing interests

The authors declare that they have no competing interests.

## Authors' contributions

YF conceived of the study. All authors helped to draft the manuscript and all authors read and approved the final manuscript.

## Pre-publication history

The pre-publication history for this paper can be accessed here:

http://www.biomedcentral.com/1471-2458/11/933/prepub

## Supplementary Material

Additional file 1 **Notification of Expedited Review Approval**. Click here for file

Additional file 2 **Notice of Award**. Click here for file
