# Supplementary material for: The mPED randomized controlled clinical trial: applying mobile persuasive technologies to increase physical activity in sedentary women protocol
Source: BMC Public Health. 2011 Dec 14;11:933. doi: 10.1186/1471-2458-11-933 (PMC3295748; doi:10.1186/1471-2458-11-933)
Supplement: Additional file 1 — Notification of Expedited Review Approval. [file 1471-2458-11-933-S1.PDF]

UCSF
University of California
San Francisco

Human Research Protection Program
Committee on Human Research

Notification of Expedited Review Approval

Principal Investigator	*Co-Principal Investigator*
Yoshimi Fukuoka, PhD, RN	

Type of Submission: Submission Correction for Modification Form
Study Title: Applying Mobile-Persuasive Technologies to Increase Physical Activity in Women

IRB #: 10-04566
Reference #: 027869

Committee of Record: Laurel Heights Panel

Study Risk Assignment: Minimal

Approval Date: 9/23/2011 **Expiration Date:** 02/14/2012

All changes to a study must receive CHR approval before they are implemented. Follow the modification request instructions. The only exception to the requirement for prior CHR review and approval is when the changes are necessary to eliminate apparent immediate hazards to the subject (45 CFR 46.103.b.4, 21 CFR 56.108.a). In such cases, report the actions taken by following these instructions.

Expiration Notice: The iMedRIS system will generate an email notification eight weeks prior to the expiration of this study's approval. However, it is your responsibility to ensure that an application for continuing review approval has been submitted by the required time. In addition, you are required to submit a study closeout report at the completion of the project.

Approved Documents: To obtain a list of documents that were approved with this submission, follow these steps: Go to My Studies and open the study – Click on Submissions History – Go to Completed Submissions – Locate this submission and click on the Details button to view a list of submitted documents and their outcomes.

For a list of all currently approved documents, follow these steps: Go to My Studies and open the study – Click on Informed Consent to obtain a list of approved consent documents and Other Study Documents for a list of other approved documents.

San Francisco Veterans Affairs Medical Center (SFVAMC): If the SFVAMC is engaged in this research, you must secure approval of the VA Research & Development Committee in addition to CHR approval and follow all applicable VA and other federal requirements. The CHR website has more information.
