# Supplementary material for: The mPED randomized controlled clinical trial: applying mobile persuasive technologies to increase physical activity in sedentary women protocol
Source: BMC Public Health. 2011 Dec 14;11:933. doi: 10.1186/1471-2458-11-933 (PMC3295748; doi:10.1186/1471-2458-11-933)
Supplement: Additional file 2 — Notice of Award. [file 1471-2458-11-933-S2.PDF]

RESEARCH **Issue Date:** 06/17/2011
Department of Health and Human Services
National Institutes of Health
NATIONAL HEART, LUNG, AND BLOOD INSTITUTE

Grant Number: 5R01HL104147-02

Principal Investigator(s):
YOSHIMI FUKUOKA, PHD

Project Title: Applying Mobile-Persuasive Technologies to Increase Physical Activity in Women

Joyce Abe
UCSF- Institute for Health and Aging
3333 California Street
Suite 340
San Francisco, CA 94118

Award e-mailed to: cgrasteam@ucsf.edu

Budget Period: 07/01/2011 – 06/30/2012
Project Period: 08/14/2010 – 06/30/2015

Dear Business Official:

The National Institutes of Health hereby awards a grant in the amount of $671,037 (see "Award Calculation" in Section I and "Terms and Conditions" in Section III) to UNIVERSITY OF CALIFORNIA SAN FRANCISCO in support of the above referenced project. This award is pursuant to the authority of 42 USC 241 42 CFR 52 and is subject to the requirements of this statute and regulation and of other referenced, incorporated or attached terms and conditions.

Acceptance of this award including the "Terms and Conditions" is acknowledged by the grantee when funds are drawn down or otherwise obtained from the grant payment system.

Each publication, press release or other document that cites results from NIH grant-supported research must include an acknowledgment of NIH grant support and disclaimer such as "The project described was supported by Award Number R01HL104147 from the National Heart, Lung, And Blood Institute. The content is solely the responsibility of the authors and does not necessarily represent the official views of the National Heart, Lung, And Blood Institute or the National Institutes of Health."

Award recipients are required to comply with the NIH Public Access Policy. This includes submission to PubMed Central (PMC), upon acceptance for publication, an electronic version of a final peer-reviewed, manuscript resulting from research supported in whole or in part, with direct costs from National Institutes of Health. The author's final peer-reviewed manuscript is defined as the final version accepted for journal publication, and includes all modifications from the publishing peer review process. For additional information, please visit http://publicaccess.nih.gov/.

Award recipients must promote objectivity in research by establishing standards to ensure that the design, conduct and reporting of research funded under NIH-funded awards are not biased by a conflicting financial interest of an Investigator. Investigator is defined as the Principal Investigator and any other person who is responsible for the design, conduct, or reporting of NIH-funded research or proposed research, including the Investigator's spouse and dependent children. Awardees must have a written administrative process to identify and manage financial conflict of interest and must inform Investigators of the conflict of interest policy and of the Investigators' responsibilities. Prior to expenditure of these awarded funds, the Awardee must report to the NIH Awarding Component the existence of a conflicting interest and within 60 days of any new conflicting interests identified after the initial report. Awardees must comply with these and all other aspects of 42 CFR Part 50, Subpart F. These requirements also apply to subgrantees, contractors, or collaborators engaged by the Awardee under this award. The NIH website http://grants.nih.gov/grants/policy/coi/index.htm provides additional information.

NIH NGA R | Version: 396 - 01/20/2011 21:06:23 | Generated on: 06/17/2011 00:27:58

If you have any questions about this award, please contact the individual(s) referenced in Section IV.

Sincerely yours,

David Ruane
Grants Management Officer
NATIONAL HEART, LUNG, AND BLOOD INSTITUTE

Additional information follows

SECTION I – AWARD DATA – 5R01HL104147-02

Award Calculation (U.S. Dollars)

**Federal Direct Costs**	$436,671
**Federal F&A Costs**	$234,366
**Approved Budget**	$671,037
**Federal Share**	$671,037
**TOTAL FEDERAL AWARD AMOUNT**	$671,037
**AMOUNT OF THIS ACTION (FEDERAL SHARE)**	$671,037

SUMMARY TOTALS FOR ALL YEARS		
YR	THIS AWARD	CUMULATIVE TOTALS
2	$671,037	$671,037
3	$691,002	$691,002
4	$676,273	$676,273
5	$656,407	$656,407

Recommended future year total cost support, subject to the availability of funds and satisfactory progress of the project

Fiscal Information:

**CFDA Number:**	93.837
**EIN:**	1946036493A6
**Document Number:**	RHL104147A
**Fiscal Year:**	2011

IC	CAN	2011	2012	2013	2014
HL	8475146	$671,037	$691,002	$676,273	$656,407

Recommended future year total cost support, subject to the availability of funds and satisfactory progress of the project

NIH Administrative Data:
PCC: HHCP N / **OC**: 414E / **Processed**: RUANED 06/16/2011

SECTION II – PAYMENT/HOTLINE INFORMATION – 5R01HL104147-02

For payment and HHS Office of Inspector General Hotline information, see the NIH Home Page at http://grants.nih.gov/grants/policy/awardconditions.htm

SECTION III – TERMS AND CONDITIONS – 5R01HL104147-02

This award is based on the application submitted to, and as approved by, NIH on the above-titled project and is subject to the terms and conditions incorporated either directly or by reference in the following:

a. The grant program legislation and program regulation cited in this Notice of Award.
b. Conditions on activities and expenditure of funds in other statutory requirements, such as those included in appropriations acts.
c. 45 CFR Part 74 or 45 CFR Part 92 as applicable.
d. The NIH Grants Policy Statement, including addenda in effect as of the beginning date of the budget period.
e. This award notice, INCLUDING THE TERMS AND CONDITIONS CITED BELOW.

(See NIH Home Page at 'http://grants.nih.gov/grants/policy/awardconditions.htm' for certain references cited above.)

This institution is a signatory to the Federal Demonstration Partnership (FDP) Phase V Agreement which requires active institutional participation in new or ongoing FDP demonstrations and pilots.

NIH NGA R | Version: 396 - 01/20/2011 21:06:23 | Generated on: 06/17/2011 00:27:58

An unobligated balance may be carried over into the next budget period without Grants Management Officer prior approval.

This grant is subject to Streamlined Noncompeting Award Procedures (SNAP).

This award is subject to the requirements of 2 CFR Part 25 for institutions to receive a Dun & Bradstreet Universal Numbering System (DUNS) number and maintain an active registration in the Central Contractor Registration. Should a consortium/subaward be issued under this award, a DUNS requirement must be included. See http://grants.nih.gov/grants/policy/awardconditions.htm for the full NIH award term implementing this requirement and other additional information.

This award may be subject to the Transparency Act subaward and executive compensation reporting requirements of 2 CFR Part 170. See http://grants.nih.gov/grants/policy/awardconditions.htm for the full NIH award term implementing this requirement and additional award applicability information.

In accordance with P.L. 110-161, compliance with the NIH Public Access Policy is now mandatory. For more information, see NOT-OD-08-033 and the Public Access website: http://publicaccess.nih.gov/.

This award provides support for one or more clinical trials. By law (Title VIII, Section 801 of Public Law 110-85), the “responsible party” must register “applicable clinical trials” on the ClinicalTrials.gov Protocol Registration System Information Website. NIH encourages registration of all trials whether required under the law or not. For more information, see http://grants.nih.gov/ClinicalTrials_fdaaa/

Treatment of Program Income:
Additional Costs

SECTION IV – HL Special Terms and Conditions – 5R01HL104147-02

NHLBI OPERATING GUIDELINES
Funding of this award is in accordance with the NHLBI FY 2011 Operating Guidelines, which can be found at: http://www.nhlbi.nih.gov/funding/policies/operguid.htm This Non-Competing award has been made at a level below that indicated in the previous Notice of Award.

Future year commitments if any, will reflect all adjustments per FY2011 guidelines.

PERSONNEL COSTS
The Consolidated Appropriations Act, 2010 Public Law 111-117, restricts the amount of direct salary of an individual under an NIH grant to Executive Level I of the Federal Executive Pay scale. The Office of Personnel Management has recently released new salary levels for the Executive Pay scale http://grants.nih.gov/grants/guide/notice-files/NOT-OD-10-041.html. The Executive Level I annual salary rate was $196,700 for the period January 1 through December 31, 2009. Effective January 1, 2010, the Executive Level I salary level increased to $199,700. For a historical record of the salary cap, including effective dates see: http://grants.nih.gov/grants/policy/salcap_summary.htm No salary increases will be provided on this grant for the remainder of this project period. The grantee institution has the authority to rebudget funds within the total costs awarded for this grant to accommodate the new salary cap.

NHLBI ADJUSTMENTS FOR SALARY BASED AWARDS
Salary funds provided on NHLBI research grants will be adjusted if investigators receive career-type salary based awards. Examples of such awards include the Independent Scientist Award (K02, formerly K04), and other similar awards as described in the November 1993 Circulation article (Vol. 88, No. 5, Part 1). In the event that such an award is made for an investigator receiving salary support from an NHLBI grant, the Institute must be informed in writing within 30 days from the start date of the award so that any required adjustment can be made.

KEY PERSONNEL
In addition to the PI, any absence, replacement, or substantial reduction in effort of the following individual(s) below, requires written prior approval of the National Institutes of Health awarding component.
Dr. John Canny

Dr. Kathleen Dracup
Dr. Eric Vittinghoff

CONSORTIUM/CONTRACTUAL COSTS
This award includes funds awarded for consortium activity with The Regents of the University of California,Berkeley. The grantee, as the direct and primary recipient of NIH grant funds, is accountable to NIH for the performance project, the appropriate expenditures of grant funds by all parties, and all other obligations of the grantee, as specified in the NIH Grants Policy Statement at: http://grants.nih.gov/grants/policy/nihgps_2010/nihgps_ch15.htm#consortium_agreements. In general, the requirements that apply to the grantee, including the intellectual property requirements also apply to consortium participant (s).

STAFF CONTACTS

The Grants Management Specialist is responsible for the negotiation, award and administration of this project and for interpretation of Grants Administration policies and provisions. The Program Official is responsible for the scientific, programmatic and technical aspects of this project. These individuals work together in overall project administration. Prior approval requests (signed by an Authorized Organizational Representative) should be submitted in writing to the Grants Management Specialist. Requests may be made via e-mail.

Grants Management Specialist: David Ruane
Email: ruaned@nhlbi.nih.gov **Phone**: 301-435-0150 **Fax**: 301-451-5462

Program Official: Denise Bonds
Email: bondsde@mail.nih.gov **Phone**: 301-435-0379

SPREADSHEET SUMMARY
GRANT NUMBER: 5R01HL104147-02

INSTITUTION: UNIVERSITY OF CALIFORNIA SAN FRANCISCO

Facilities and Administrative Costs	*Year 2*	*Year 3*	*Year 4*	*Year 5*
F&A Cost Rate 1	54.5%	54.5%	54.5%	54.5%
F&A Cost Base 1	$430,030	$440,152	$430,405	$417,328
F&A Costs 1	$234,366	$239,883	$234,571	$227,444

NIH NGA R | Version: 396 - 01/20/2011 21:06:23 | Generated on: 06/17/2011 00:27:58
